# How are plant and fungal communities linked to each other in belowground ecosystems? A massively parallel pyrosequencing analysis of the association specificity of root-associated fungi and their host plants

**DOI:** 10.1002/ece3.706

**Published:** 2013-08-02

**Authors:** Hirokazu Toju, Hirotoshi Sato, Satoshi Yamamoto, Kohmei Kadowaki, Akifumi S Tanabe, Shigenobu Yazawa, Osamu Nishimura, Kiyokazu Agata

**Affiliations:** 1 Graduate School of Global Environmental Studies, Kyoto UniversityKyoto, 606-8501, Japan; 2 Graduate School of Human and Environmental Studies, Kyoto UniversityKyoto, 606-8501, Japan; 3 Graduate School of Science, Kyoto UniversityKyoto, 606-8502, Japan

**Keywords:** Common mycorrhizal network, endophytes, metagenomics, mycorrhizae, network theory, plant communities

## Abstract

In natural forests, hundreds of fungal species colonize plant roots. The preference or specificity for partners in these symbiotic relationships is a key to understanding how the community structures of root-associated fungi and their host plants influence each other. In an oak-dominated forest in Japan, we investigated the root-associated fungal community based on a pyrosequencing analysis of the roots of 33 plant species. Of the 387 fungal taxa observed, 153 (39.5%) were identified on at least two plant species. Although many mycorrhizal and root-endophytic fungi are shared between the plant species, the five most common plant species in the community had specificity in their association with fungal taxa. Likewise, fungi displayed remarkable variation in their association specificity for plants even within the same phylogenetic or ecological groups. For example, some fungi in the ectomycorrhizal family Russulaceae were detected almost exclusively on specific oak (*Quercus*) species, whereas other Russulaceae fungi were found even on “non-ectomycorrhizal” plants (e.g., *Lyonia* and *Ilex*). Putatively endophytic ascomycetes in the orders Helotiales and Chaetothyriales also displayed variation in their association specificity and many of them were shared among plant species as major symbionts. These results suggest that the entire structure of belowground plant–fungal associations is described neither by the random sharing of hosts/symbionts nor by complete compartmentalization by mycorrhizal type. Rather, the colonization of multiple types of mycorrhizal fungi on the same plant species and the prevalence of diverse root-endophytic fungi may be important features of belowground linkage between plant and fungal communities.

## Introduction

Under natural conditions, several hundred fungal species are associated with plant roots within forests (Ishida et al. 2007; Öpik et al. 2009; Jumpponen et al. 2010). These fungi are considered to be essential agents that determine the composition of plant communities (Booth 2004; Nara and Hogetsu 2004; Peay et al. 2010). For example, mycorrhizal fungi facilitate the soil nutrient acquisition of plants (Smith and Read 2008) and thereby enhance the competitive ability of their specific hosts in local communities (Nara 2006). Likewise, phylogenetically diverse fungal root endophytes not only promote the growth of plants but also enhance the pathogen resistance of their hosts (Upson et al. 2009; Newsham 2011), while some of them are known to negatively affect the fitness of host plants (Reininger and Sieber 2012). Thus, ecologically and phylogenetically diverse fungi differentially interact with plant species in the wild, potentially playing important roles in the dynamics of forest ecosystems (Klironomos 1999, 2003; Fukami and Nakajima 2013).

In natural forests, importantly, associations between plants and their fungal symbionts are generally “non-random” (Davison et al. 2011; Chagnon et al. 2012; Montesinos-Navarro et al. 2012). That is, whereas plants select for their fungal symbionts (Kiers et al. 2011), root-associated fungi display preference for host plant species (Bruns et al. 2002; Tedersoo et al. 2008; Walker et al. 2011). Many previous studies have revealed the host preference of tens or hundreds of fungal species in natural forests (Kennedy et al. 2003; Tedersoo et al. 2008; Davison et al. 2011). Of particular interest is the study by Öpik et al. (2009), which investigated the composition of an arbuscular mycorrhizal fungal community by analyzing the roots of 10 plant species occurring in an Estonian boreonemoral forest. This community ecological analysis, based on 454 pyrosequencing (Margulies et al. 2005), revealed that several arbuscular mycorrhizal fungal taxa were shared among the 10 plant species, but many other taxa were detected only from some of the potential host species. These kind of community ecological studies provided a basis for determining how variation in the host preference of root-associated fungi influences the dominance of specific host plants or the coexistence of diverse plant species in natural forests (Klironomos 1999, 2003).

To date, most studies of root-associated fungal communities have focused on particular functional or phylogenetic groups of fungi (e.g., Öpik et al. 2009). However, diverse types of root-associated fungi can be hosted in a wild plant community (Dickie et al. 2004; Toju et al. 2013). This within-community diversity of root-associated fungi is important because many recent studies have reported “non-typical” plant–fungal associations that are not classified into the conventional categories of mycorrhizal symbiosis (Dickie et al. 2004; Curlevski et al. 2009). Examples of these associations include ericoid mycorrhizal fungi on ectomycorrhizal plants (Chambers et al. 2008; Grelet et al. 2009), ectomycorrhizal fungi on ericoid mycorrhizal plants (Vohník et al. 2007), arbuscular mycorrhizal fungi on ectomycorrhizal plants (Dickie et al. 2001; Mcguire et al. 2008; Yamato et al. 2008) and ectomycorrhizal fungi on arbuscular mycorrhizal plants (Murata et al. 2012). These studies suggest that mycorrhizal interactions are more complex and flexible than was previously recognized. In addition, recent studies have shown that diverse clades of endophytic fungi commonly colonize plant roots with mycorrhizal fungi in temperate and Arctic regions, thereby further complicating the belowground plant–fungal associations (Newsham 2011; Toju et al. 2013). Given these facts, studies of plant–fungal associations need to be expanded to cover the entire community, wherein multiple types of fungi (e.g., ectomycorrhizal, arbuscular mycorrhizal, and root-endophytic fungi) and all of their plant hosts are included.

The aim of this study was to investigate the entire structure of belowground plant–fungal associations by targeting all phylogenetic groups of fungi and their hosts. In a temperate boreonemoral forest in Japan, we collected root samples of 33 plant species and analyzed the species-rich community of root-associated fungi based on 454 pyrosequencing of internal transcribed spacer (ITS) sequences. As in many other fungal community analyses based on molecular data, the presence of a fungal ITS sequence in a root sample represents a root–hyphal connection, but not necessarily a mutualistic plant–fungal interaction (Caruso et al. 2012). Thus, the high-throughput pyrosequencing data were used to evaluate the specificity of root–hyphal connections (hereafter, association specificity), which reflected the partner preference of plants and fungi, but could be affected not only by mutualistic interactions but also by commensalistic or neutral interactions. On the basis of the analysis, we examined whether or not the conventional classification of mycorrhizal symbiosis could fully depict the entire structure of belowground plant–fungal associations. Overall, this study suggests that more ecological studies are necessary to understand the diversity and complexity of belowground associations between root-associated fungi and their host plants.

## Material and Methods

### Sampling and DNA extraction

Roots were sampled from a temperate secondary forest on Mt. Yoshida, Kyoto, Japan (35°02′N, 135°47′E; parent material = chert), from 1 July to 7 July 2010. At the study site, a deciduous oak, *Quercus serrata*, and an evergreen oak, *Quercus glauca*, are the dominant tree species, whereas evergreen trees such as *Ilex pedunculosa* (Aquifoliaceae) and *Pinus densiflora* (Pinaceae) and deciduous trees such as *Lyonia ovalifolia* (Ericaceae) and *Prunus grayana* (Rosaceae) co-occur. A 59 m × 15 m plot was established and sampling positions were set at 1-m intervals (i.e., 60 rows × 16 columns = 960 sampling positions). At each sampling position, we dug plant roots from the upper part of the A horizon (3 cm below the soil surface) and then sampled two approximately 2-cm segments of terminal root. As the sampling was indiscriminate in terms of root morphology and mycorrhizal type, our samples included roots potentially colonized not only by mycorrhizal fungi but also by diverse root-endophytic fungi. In addition, because of the sampling design, the root samples were considered to approximately represent the belowground biomass composition of the plant community at the study site. The root samples were immediately preserved in absolute ethanol and stored at −25°C in the laboratory.

### DNA extraction, PCR, and pyrosequencing

One terminal root was randomly selected from each of the 960 sampling positions. All soil was carefully removed from the samples by placing them in 70% ethanol with 1-mm zirconium balls and then shaking the sample tubes 15 times per second for 2 min using a TissueLyser II (Qiagen, Venlo, The Netherlands) (Toju et al. 2013). The washed root was frozen at –25°C and then pulverized by shaking with 4-mm zirconium balls 20 times per second for 3 min using a TissueLyser II. Plant and fungal DNA was extracted from each root sample by a cetyl trimethyl ammonium bromide (CTAB) method as described by Sato and Murakami (2008).

We sequenced host plant chloroplast *rbcL* and fungal ITS sequences based on a tag-encoded massively parallel pyrosequencing analysis (Toju et al. 2013). For each root sample, plant *rbcL* sequences were amplified using the primers rbcL_rvF (5′-CCA MAA ACR GAR ACT AAA GC-3′) and rbcL_R1 (5′-CGR TCY CTC CAR CGC AT-3′) with a buffer system of Ampdirect Plus (Shimadzu Corp., Kyoto, Japan) and BIOTAQ HS DNA Polymerase (Bioline, London, U.K.). Polymerase chain reaction (PCR) was conducted using a temperature profile of 95°C for 10 min, followed by 30 cycles at 94°C for 20 sec, 50°C for 30 sec, 72°C for 30 sec, and a final extension at 72°C for 7 min. The PCR product of each root sample was subjected to a second PCR amplification of a 0.5-kb *rbcL* gene fragment using the rbcL_rvF primer fused with the 454 pyrosequencing Adaptor A (5′-CCA TCT CAT CCC TGC GTG TCT CCG ACT CAG-3′) and the 8-mer molecular ID (Hamady et al. 2008) of each sample, and the reverse primer rbcL_R2 (5′-CCY AAT TTT GGT TTR ATR GTA C-3′) fused with the 454 Adaptor B (5′-CCT ATC CCC TGT GTG CCT TGG CAG TCT CAG-3′). The second PCR was conducted with a buffer system of Taq DNA Polymerase with Standard Taq Buffer (New England BioLabs, Ipswich, MA) under a temperature profile of 95°C for 1 min, followed by 40 cycles at 94°C for 20 sec, 50°C for 30 sec, 72°C for 30 sec, and a final extension at 72°C for 7 min.

For the analysis of fungal ITS sequences, the entire ITS region was amplified using the fungus-specific high-coverage primer ITS1F_KYO2 (Toju et al. 2012) and the universal primer ITS4 (White et al. 1990). The PCR product of each root sample was subjected to a second PCR step targeting the ITS2 region using the universal primer ITS3_KYO2 (Toju et al. 2012) fused with the 454 Adaptor A and each sample-specific molecular ID, and the reverse universal primer ITS4 fused with the 454 Adaptor B. The first and second PCR steps for the ITS region were conducted using the same buffer systems and temperature profiles as those of *rbcL*.

The *rbcL* and ITS amplicons from the second PCR step were subjected to pyrosequencing. To obtain more than 100 ITS reads per sample on average, the first 480 and the second 480 samples were sequenced separately using a GS Junior sequencer (Roche, Basel, Switzerland). The *rbcL* and ITS amplicons from the first 480 root samples were pooled and purified using ExoSAP-IT (GE Healthcare, Little Chalfont, Buckinghamshire, U.K.) and a QIAquick PCR Purification Kit (Qiagen). The sequencing of the first 480 samples was conducted according to the manufacturer's instructions. The amplicons of the remaining 480 samples were pooled and purified, and then sequenced in the second run.

### Assembling of pyrosequencing reads

Hereafter, the bioinformatics pipeline is described, referring to the criteria for the standardized description of next-generation sequencing methods (Nilsson et al. 2011). In the pyrosequencing, 95,438, and 97,932 reads were obtained for the first and second runs, respectively (DDBJ Sequence Read Archive: DRA000935). For the pyrosequencing reads, the trimming of low-quality 3′ tails was conducted with a minimum quality value of 27. After the trimming step, 84,339 (15,017 *rbcL* and 69,322 ITS reads) and 84,040 (16,233 *rbcL* and 67,807 ITS reads) reads for the first and second runs, respectively, passed the filtering process in which *rbcL* and ITS reads with shorter than 150 bp excluding forward primer and molecular ID positions were discarded. *RbcL* and ITS reads were recognized by the primer position sequences and analyzed separately. For each gene, pyrosequencing reads were sorted based on combinations of the sample-specific molecular IDs and pyrosequencing runs (i.e., 480 IDs × 2 runs = 960 samples). Molecular ID and forward primer sequences were removed before the assembly process. Denoising of sequencing data was performed based on the assembly analysis detailed below (cf. Li et al. 2012).

For the analysis of the host plant *rbcL* gene, reads were assembled using Assams-assembler v0.1.2012.05.24 (Tanabe 2012a; Toju et al. 2013), which is a highly parallelized extension of the Minimus assembly pipeline (Sommer et al. 2007). Reads in each sample were assembled with a minimum cutoff similarity of 97% to remove pyrosequencing errors, and the consensus *rbcL* gene sequence of each root sample was then obtained. After the elimination of possible chimeras using UCHIME v4.2.40 (Edgar et al. 2011) with a minimum score of 0.1 to report a chimera, the consensus sequences for root samples (within-sample consensus sequences) were further assembled across samples with a minimum similarity setting of 99.8%. These consensus sequences (among-sample consensus sequences) were compared to the reference *rbcL* sequences in the NCBI nucleotide database (http://www.ncbi.nlm.nih.gov/) to identify the host plant species of each root sample.

In the analysis of the fungal ITS2 region, the 137,129 (69,322 in the first run and 67,807 in the second run) reads were subjected to the detection and removal of chimeras using UCHIME after obtaining within-sample consensus sequences with a minimum cutoff similarity of 97%. Of the 137,129 ITS reads, 1598 reads were discarded as chimeras, leaving a total of 135,531 reads.

The within-sample consensus sequences represented by the 135,531 reads were assembled across samples. Given that fungal ITS sequences sometimes show >3% intraspecific variation (Nilsson et al. 2008), the minimum cutoff similarity of the among-sample assembling process was set to 95% in Assams-assembler. The resulting consensus sequences represented fungal operational taxonomic units (OTUs; Data S1). Of the 135,531 reads, 537 were excluded as singletons. Samples with fewer than 20 high-quality reads were eliminated, leaving 834 root samples. On average, 152.2 (SD = 47.9) ITS reads were obtained for each sample (Data S2).

### Molecular identification of fungi

To systematically infer the taxonomy of respective OTUs, local BLAST databases were prepared based on the “nt” database downloaded from the NCBI ftp server (http://www.ncbi.nlm.nih.gov/Ftp/) on 11 May 2012. Molecular identification of OTUs was conducted through local BLAST searches using Claident v0.1.2012.05.21 (Tanabe 2012b; Toju et al. 2013), which integrated BLAST+ (Camacho et al. 2009) and NCBI taxonomy-based sequence identification engines based on the lowest common ancestor algorithm (Huson et al. 2007). Based on the molecular identification, OTUs were classified into ectomycorrhizal fungi, arbuscular mycorrhizal fungi, and fungi with unknown nutritional modes (Data S3). To screen for ectomycorrhizal fungi, we referred to a review by Tedersoo et al. (2010).

### Community data matrices

For each of the 834 samples from which both *rbcL* and ITS sequences were successfully obtained, the presence/absence of respective fungal OTUs was evaluated using the following process. Only OTUs with more than 5% of sample total reads were regarded as being present in a sample to reduce variance in *α*-diversity among samples that results from variance in sequencing effort (i.e., variance in the number of sequencing reads among samples: Data S2; cf. Gihring et al. 2012). From this process, a binary matrix depicting the presence or absence of OTUs in each sample was obtained (Data S4: hereafter, “sample-level” matrix). In the matrix, the plant species information of each root sample was supplied based on the *rbcL* data (see above).

The “sample-level” data matrix was used to construct a matrix representing associations between plant species and fungal OTUs (Data S5: hereafter, “plant × fungal” matrix). In the matrix, rows represented plant species and columns represented fungal OTUs. In the “plant × fungal” matrix, a value in a cell represented the number of root samples in which the focal plant–fungal association was observed (Data S5).

### Fungi shared among plant species and those unique to each plant

Based on the “plant × fungal” matrix, the number of fungal OTUs shared between species was obtained for each pair of plant species. In addition, for each plant species, the number of fungal OTUs unique to the plant or the number of fungal OTUs shared with other plant species was indicated.

### Measure of association specificity

To quantitatively evaluate the plants' association specificity for fungal OTUs, the *d′* index of the specialization of interspecific associations (Blüthgen et al. 2007) was estimated for each plant species based on the “plant × fungal” matrix (Data S5). The *d′* index measures how strongly a plant species (a fungus) deviates from a random choice of interacting fungal partners (host plant partners) available. The index ranges from 0 (extreme generalization) to 1 (extreme specialization; Blüthgen et al. 2007). The “bipartite” v1.17 package (Dormann et al. 2009) of R (http://cran.r-project.org/) was used for the analysis. The observed *d′* index values were compared with those of a randomized “plant × fungal” matrix, in which combinations of plant species and fungal OTUs were randomized with the “vaznull” model (Vázquez et al. 2007) using the bipartite package (10,000 permutations). A *d′* index higher than expected by chance indicated association specificity for fungal OTUs in a focal plant species.

In addition to the plants' association specificity for fungal OTUs, the fungal association specificity for plant species was also evaluated using the *d′* index.

### Comparison of fungal community structure between common plant species

Although the *d′* index revealed the degree of association specificity, it did not identify which plant–fungal combinations were prevalent at the study site. Thus, we conducted a further analysis of plant–fungal associations to screen for fungi preferentially associated with specific host plant species and those with a broad host range by statistically investigating how each fungal OTU was shared among the dominant plant species. For each pair of the five most common host species (Fig. S1A), we used the multinomial species classification method (i.e., CLAM test; Chazdon et al. 2011) to statistically classify fungal OTUs into the following categories: fungi common on both plants, fungi preferentially associated with either plant, and fungi that were too rare to be assigned association specificity. The CLAM analysis was performed based on the “sample-level” data matrix (Data S4) using the vegan v.2.0-2 package (Oksanen et al. 2012) of R with “supermajority” rule (Chazdon et al. 2011).

## Results

### Pyrosequencing and community data matrices

In total, we found 836 fungal OTUs excluding singletons and possible chimeras from the 834 sequenced terminal root samples (Data S2). The mean number of OTUs observed in a sample was 8.4 (SD = 4.0; see also Fig. S2A). The total number of observed OTUs increased almost linearly with increasing sample size (Fig. S2B).

Of the 836 OTUs observed, 676 (80.9%) were identified at the phylum level. Of these 676 OTUs, 438 (64.8%) were ascomycetes, 214 (31.7%) basidiomycetes, four (0.6%) were chytridiomycetes, and 20 (3.0%) were glomeromycetes (Fig. S1B). At the order level, 431 (51.6%) OTUs were identified. Among them, Agaricales (13.9%), Helotiales (12.5%), Russulales (11.1%), Hypocreales (7.2%), and Chaetothyriales (4.4%) accounted for approximately half of the identified fungal community, whereas other diverse orders were also observed at lower frequencies (Fig. S1C). At the genus level, 221 (26.4%) OTUs were identified. Of the 221 OTUs, three ectomycorrhizal genera, *Russula* (10.4%), *Cortinarius* (9.0%), and *Lactarius* (6.8%), constituted more than a quarter of the total community, whereas diverse ectomycorrhizal (e.g., *Amanita*, *Sebacina*, *Tomentella*, *Cenococcum*, *Inocybe,* and *Clavulina*), arbuscular mycoirrhizal (e.g., *Glomus* and *Gigaspora*), and nonmycorrhizal (e.g., *Trechispora*, *Mortierella*, *Mycena*, *Capronia*, *Cladophialophora,* and *Hypocrea*) genera were also detected (Fig. S1D).

Sequencing of the chloroplast *rbcL* gene revealed that the 834 terminal root samples represented 33 plant species (Fig. S1A). Among the 33 plant species, the most common were two oak species, *Q. glauca* and *Q. serrata* (Fig. S1A). Roots of a broad-leaved evergreen species(*I. pedunculosa*), a deciduous ericaceous species (*Lyonia ovalifolia*), and an evergreen pine species (*P. densifolia*) were also observed with a high frequency, and the five most common species, such as the two oak trees, comprised 80.1% of the 834 root samples (Fig. S1A).

When only the OTUs with more than 5% of the sample total reads were regarded as present in a sample, 387 OTUs were found in the “sample-level” matrix (Data S4). Of the 387 OTUs, 85 were considered to be ectomycorrhizal and 10 were arbuscular mycorrhizal (Data S3). Based on the “sample-level” matrix, a “plant × fungal” matrix was obtained (Data S5). Among the fungal OTUs in the matrix, diverse ascomycete and basidiomycete ectomycorrhizal fungi in genera including *Elaphomyces*, *Cenococcum*, *Clavulina*, *Lactarius*, *Russula,* and *Tomentella* were observed at a high frequency, while ascomycetes with unknown nutritional modes were most dominant (Table 1). Many of these poorly understood ascomycetes belonged to such orders as Helotiales and Chaetothyriales (Table 1; see also Data S3).

**Table 1:** The 15 most common fungal OTUs in the plant–fungal associations

OTU ID	*N*	Description				BLAST top-hit			
	
		Phylum	Order	Family	Genus	Description	E value	Identity	Accession
158	260	Ascomycota	Helotiales^1^			Hyaloscyphaceae sp.	3E-151	98%	JQ272392.1
636	226	Ascomycota	Helotiales			Helotiales sp.	1E-155	100%	JF273525.1
1334	112	Ascomycota	Chaetothyriales	Herpotrichiellaceae		*Cladophialophora* sp.	5E-139	93%	EU139132.1
226	65	Ascomycota	Eurotiales	Elaphomycetaceae	*Elaphomyces*^2^	*Elaphomyces decipiens*	5E-139	93%	EU837229.1
388	64	Basidiomycota	Russulales	Russulaceae	*Lactarius*^2^	*Arcangeliella camphorata*	0	96%	EU644700.1
1	60	Basidiomycota	Cantharellales	Clavulinaceae	*Clavulina*^2^	*Clavulina* sp.	0	100%	JF273519.1
1580	59	Ascomycota	Chaetothyriales	Herpotrichiellaceae	*Capronia*	*Capronia* sp.	2E-162	98%	AF284128.1
248	53	Ascomycota	–	–	*Cenococcum*^2^	*Cenococcum geophilum*	6E-153	98%	JQ711949.1
314	52	Basidiomycota	Russulales	Russulaceae^2^		*Russula japonica*	2E-162	96%	AB509603.1
1312	52	Basidiomycota	Russulales	Russulaceae	*Lactarius*^2^	*Lactarius helvus*	7E-177	93%	AY606946.1
1692	49	Ascomycota	Helotiales	Dermateaceae		Helotiales sp.	4E-159	99%	HQ260955.1
176	48	Ascomycota	Chaetothyriales	Herpotrichiellaceae		*Cladophialophora carrionii*	1E-139	93%	HM803232.1
48	44	Basidiomycota	Russulales	Russulaceae	*Russula*^2^	*Russula cerolens*	0	98%	JN681168.1
548	41	Basidiomycota	Thelephorales	Thelephoraceae	*Tomentella*^2^	*Tomentella* sp.	0	99%	JF273546.1
1046	41	Ascomycota	Helotiales			*Cryptosporiopsis* sp.	4E-100	88%	JN601680.1

The ID numbers of OTUs and the number of terminal root samples in which each fungus was observed are shown. The results of molecular identification based on Claident and manual BLAST searches are shown for each OTU.

1 Identified based on additional manual BLAST search.

2 Putatively ectomycorrhizal lineages.

### Fungi shared among plant species and those unique to each plant

**Figure 1:**
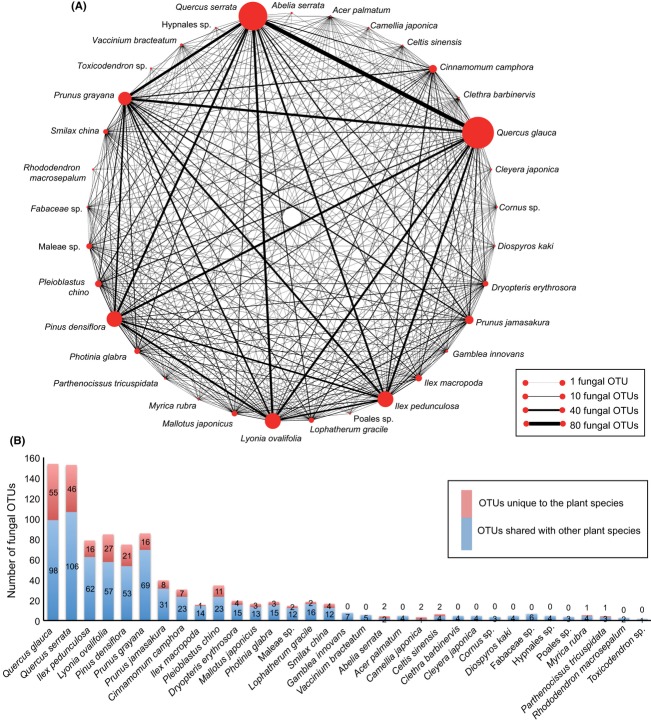
Sharing of fungal OTUs among plant species in the community. (A) The number of fungal OTUs shared among plant species. The line thickness is proportional to the number of fungal OTUs shared between each pair of plant species. The size of circles roughly represents the composition of plant species in the samples (Fig. S1A). Common plant species in the community are located away from each other so as to make it easier to grasp the number of shared fungal OTUs. (B) The number of fungal OTUs detected from each plant species. The number of OTUs identified only from a focal plant species (OTUs unique to the plant species) and that of OTUs that was detected also from plant species other than the focal one (OTUs shared with other plant species) is separately shown. Plant species are shown in the decreasing order of the number of terminal root samples (Fig. S1A).

The analysis of the “plant × fungal” matrix indicated that the plant species shared many root-associated fungal symbionts in the study forest and that there was no plant species isolated in the graph that represented the number of shared fungal OTUs (Fig. 1A). For example, 82, 40, and 40 fungal OTUs were shared between *Q. glauca* and *Q. serrata*, between *Q. glauca* and *Pinus densiflora,* and between *Q. glauca* and *P. densiflora* (Fig. 1A). Intriguingly, each of the two dominant plants shared at least one fungal OTU with all the 32 remaining plant species (Fig. 1A).

Of the 387 fungal taxa analyzed, 153 (39.5%) were detected from at least two plant species. For most plant species, the number of fungal OTUs shared with other plants exceeded that of the OTUs unique to the plant (Fig. 1B). In particular, only 18.8–35.9% of the observed fungal OTUs were unique to each of the five most common plant species (Fig. 1B).

### Measure of association specificity

**Figure 2:**
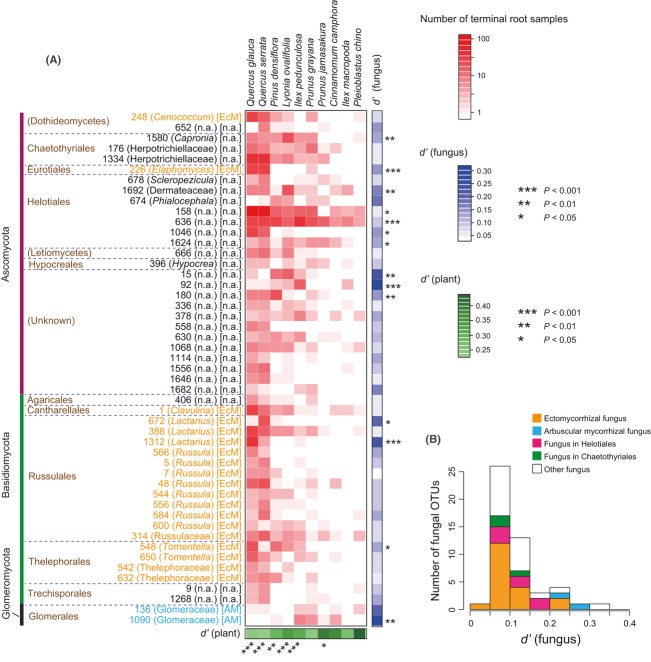
Association specificity analysis. (A) Plant × fungal matrix and the *d′* measure of association specificity. The red boxes represent the number of times (terminal root samples) in which respective plant × fungal combinations are observed. Based on the *d′* index of the specialization of interspecific associations (Blüthgen et al. 2007), association specificity of each plant species (green) and that of each fungal OTU (blue) were estimated. Results of plant species with 10 or more root samples (Fig. S1A) and the fungal OTUs that appeared in 10 or more root samples are shown. See Table S1 for *d′* measures of all the examined plants and fungi. For each OTU, genus or family name is shown in a parenthesis and mycorrhizal type in a bracket. (B) Histogram of the association specificity of fungi. Results of the fungal OTUs that appeared in 10 or more root samples are shown.

The analysis of *d′* index values revealed that the five dominant plant species displayed a significantly high association specificity for fungal OTU(s) (Fig. 2A; Table S1). In addition to these five species, *Prunus jamasakura* also displayed marginally significant association specificity (Table S1).

For fungi, a remarkable variation in association specificity was observed, even among fungi in the same phylogenetic or ecological groups (Fig. 2A, B; Table S1). For example, two ectomycorrhizal fungi in the family Russulaceae (OTUs 1312 and 672) displayed significant association specificity for plant species, whereas the remaining 10 OTUs in the same family did not (Fig. 2A). Likewise, of the two frequently observed ectomycorrhizal ascomycetes, *Elaphomyces* sp. (OTU 226) had statistically significant association specificity, whereas *Cenococcum* sp. (OTU 248) were found on diverse plant species (Fig. 2A). Ascomycetes with unknown nutritional modes displayed a high variation in the degree of association specificity within the orders Chaetothyriales and Helotiales (Fig. 2). Of the two most frequently observed arbuscular mycorrhizal OTUs, one (OTU 1090) had a statistically significant association specificity, whereas the other (OTU 136) did not (Fig. 2A). Among the fungi that appeared in 10 or more root samples, an unidentified fungus (OTU 92) and an arbuscular mycorrhizal fungus displayed the highest association specificity (Fig. 2B). Rare fungi (i.e., fungi appearing in less than 10 root samples) were detected with very low or high *d′* index values (Table S1), which preferentially appeared in the roots of common or rare plant species at the study site (Data S5). However, due to the high estimation error expected from the small sample size, the *d′* index value estimates for these rare fungi should be interpreted cautiously.

### Comparison of fungal community structure between common plant species

**Figure 3:**
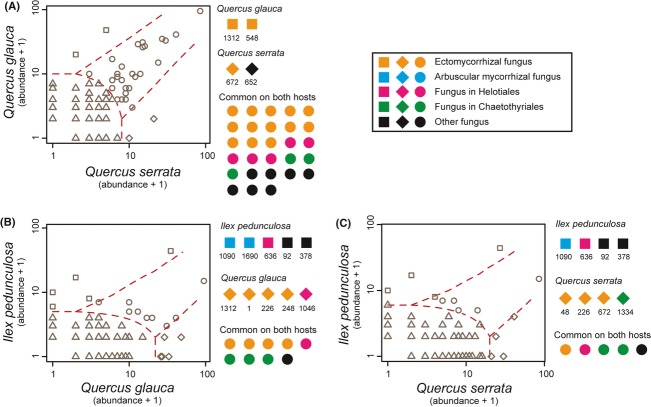
Comparison of fungal community structure between common plant species. For each pair of host plant species, a CLAM analysis (Chazdon et al. 2011) classified fungal OTUs into the following categories: fungi common on both plants (circle), fungi preferentially associated with either plant (square and diamond), and fungi that were too rare to be assigned association specificity (triangle). Results for the three most common host plants are shown (see Fig. S3 for results for other pairs of host plants). The ID numbers of fungal OTUs with significant host preference are indicated under the symbols. (A) *Quercus glauca* versus *Quercus serrata*. (B) *Q. glauca* versus *Ilex pedunculosa*. (C) *Q. serrata* versus *I. pedunculosa*.

Based on a CLAM analysis, a statistical screening for fungal OTUs preferentially associated with specific plant species was undertaken for each pair of the five most common plant species (Fig. 3; Table S2). For example, an ectomycorrhizal basidiomycete in the genus *Lactarius* (OTU 1312) consistently displayed association specificity for *Q. glauca* in all the pairs examined, whereas another *Lactarius* species (OTU 672) preferred *Q. serrata* (Figs. 3 and S3; Table S2). Likewise, an arbuscular mycorrhizal fungus (OTU 1090) consistently preferred *I. pedunculosa* in all the examined host plant pairs (Figs. 3 and S3; Table S2). An ectomycorrhizal ascomycete in the genus *Elaphomyces* (OTU 226) was commonly found associated with the two *Quercus* species (Fig. 2; Table S2) and displayed a significant association specificity for the two host species (Figs. 3 and S3).

The CLAM analysis also indicated that 28 OTUs were statistically classified as fungal taxa common to the two dominant *Quercus* species (Fig. 3). Of the 28 common taxa, 13 (46.4%) were ectomycorrhizal fungi, whereas five (17.9%) were Helotiales and three (10.7%) were Chaetothyriales (Fig. S3; Table S2). The two oak species shared ectomycorrhizal fungi with other dominant plant species, especially *P. densiflora* and *L. ovalifolia* (Figs. 3 and S3).

## Discussion

Through the massively parallel pyrosequencing analysis, we revealed the diversity and association specificity of root-associated fungi and their host plants in an oak-dominated temperate forest. Our findings can be summarized as follows. First, diverse ectomycorrhizal ascomycete and basidiomycete taxa such as *Elaphomyces*, *Cenococcum*, *Clavulina*, *Lactarius*, *Russula,* and *Tomentella* were common within the fungal community, whereas the most dominant root-associated fungal taxa were possibly root-endophytic ascomycetes of the orders Helotiales and Chaetothyriales (Table 1). Second, any two plant species studied here hosted at least one common fungal symbiont on their roots (Fig. 1). Of the fungal OTUs observed from the roots of the five most common plant species (Fig. S1A), 64.1–81.2% were hosted by multiple plant species (Fig. 1). Third, the five most common plant species in the study site and root-associated fungi in various phylogenetic/ecological groups displayed statistically significant association specificity (Figs. 3 and S3; Table 1). The *d′* index (Fig. 2; Table S1) and a CLAM analysis (Figs. 3 and S3; Table S2) indicated that the degree of association specificity varied among fungal taxa, even within the same phylogenetic or ecological group of root-associated fungi.

### Sharing of fungal taxa within the plant community

Although plants in the study forest shared up to 82 fungal taxa with other plant species (Fig. 1), the five dominant plant species in the community displayed statistically significant association specificity for root-associated fungi (Fig. 2A). The presence of association specificity for fungal symbionts per se is consistent with the commonly accepted view that plant species can be divided into several categories in terms of mycorrhizal symbiosis (Smith and Read 2008). Based on the conventional classification of mycorrhizal symbiosis, *Quercus* and *Pinus* species are regarded as ectomycorrhizal (Tedersoo et al. 2010), *I. pedunculosa* is regarded as arbuscular mycorrhizal (Yamato et al. 2008), and *L. ovalifolia* is regarded as ericoid mycorrhizal (Straker 1996). However, given the fact that several ectomycorrhizal fungal OTUs colonized all the five dominant plant species and did not show statistically significant association specificity for plant species (e.g., OTUs 1, 388 and 314; Figs. 3 and S3; Table S2), the structure of the real plant root–associated fungal symbiosis is likely to be more complicated than was previously considered.

The existence of root-hyphal connections that do not fall under the conventional classification of mycorrhizal symbiosis is supported also by the previous findings that multiple types of mycorrhizal fungi can colonize the same host plant species (Dickie et al. 2004; Curlevski et al. 2009). Those studies showed that both arbuscular mycorrhizal and ectomycorrhizal fungi or both ericoid mycorrhizal and ectomycorrhizal fungi were frequently detected on the same plant species in natural forests (Dickie et al. 2001; Chambers et al. 2008; Mcguire et al. 2008; Yamato et al. 2008). Taking into account these facts, this study further suggests that plants' associations with multiple types of mycorrhizal fungi can be usual rather than exceptional in natural environments. However, as this study entirely depended on molecular data, fungal species whose hyphae were merely adhering to nonhost plant roots might be detected in the analysis. Therefore, further histological and physiological studies are necessary to understand the prevalence and ecological consequence of root colonization by multiple types of fungi (cf. Caruso et al. 2012).

This study also indicated that many ascomycetes with unknown nutritional modes, mostly in the orders Helotiales and Chaetothyriales (Figs. 2 and 3; Table 1), were involved in belowground plant–fungal association. Although many studies have suggested the potential beneficial effects of “root-endophytic” ascomycetes on plant hosts (Upson et al. 2009; Newsham 2011), most studies on belowground plant–fungal interactions have paid little attention to those “non-mycorrhizal” fungi (Mandyam and Jumpponen 2005; Mandyam et al. 2012). This study indicated that these putatively “non-mycorrhizal” (or endophytic) ascomycetes could be commonly involved in plant root–associated fungal interactions (Figs. 2 and 3; Table 1).

### Variations in the association specificity of fungi

From a mycological perspective, our analysis has revealed remarkable variation in association specificity for plants among fungi belonging to the same phylogenetic or ecological groups (Figs. 2 and 3). Within-group variability in association specificity for plant species has been reported in recent high-throughput DNA barcoding studies on ectomycorrhizal or arbuscular mycorrhizal fungi (Ishida et al. 2007; Tedersoo et al. 2008; Öpik et al. 2009). By expanding the targets of such community ecological analyses, we have identified a method to quantitatively compare the degree of association specificity among fungi in the same or different phylogenetic/ecological groups.

For ectomycorrhizal fungi, we found that *Lactarius* OTUs displayed association specificity for one of the two *Quercus* species (i.e., OTU 1312 on *Q. glauc*a and OTU 672 on *Q. serrata*), whereas many other Russulaceae fungi were identified on a broader range of host plant species (Figs. 3 and S3; Table S2). This indicates that the degree of association specificity varies even within a phylogenetic group of ectomycorrhizal fungi. As shown in the analysis, ectomycorrhizal fungi in the same genus or family can have specificity for plants not only at the host family or genus level (Ishida et al. 2007; Tedersoo et al. 2008) but also at the species level.

Although the dominance of ectomycorrhizal plant species in the community (Fig. S1A) precluded thorough statistical testing of the association specificity of arbuscular mycorrhizal fungi, the fungal ecotype indicated some variation in association specificity (Fig. 2; Tables S1 and S2). This result was consistent with the findings of a recent pyrosequencing study, in which arbuscular mycorrhizal fungi in a forest showed varying degrees of host preference (Öpik et al. 2009). The host range of root-endophytic ascomycetes has also been recognized as broad (Knapp et al. 2012; Mandyam et al. 2012), but this study revealed considerable variation in association specificity within Helotiales and Chaetothyriales (Fig. 2).

### Conclusions and perspectives

This study revealed that diverse mycorrhizal and nonmycorrhizal fungal taxa were shared within the plant community of a temperate forest, whereas many plants and fungi showed specificity in terms of their association with partners. Thus, the entire structure of belowground plant–fungal associations may be depicted neither by complete compartmentalization by mycorrhizal type nor by the random sharing of hosts/symbionts. The fact that both ectomycorrhizal and arbuscular mycorrhizal fungi were detected from the same plant species (cf. Dickie et al. 2001) is intriguing, but further histological and physiological studies are necessary to understand the prevalence and ecological roles of such multiple colonization in the community (cf. Caruso et al. 2012). In addition, the prevalence of diverse root-endophytic fungi suggests that the knowledge of mycorrhizal symbiosis alone does not fully describe the roles of root-associated fungi in plant community dynamics. Future studies examining the community structure of both mycorrhizal and root-endophytic fungi will enhance our knowledge of the belowground linkage between plant and fungal communities and its ecological consequences.

## Supporting Information

**Data S1.** OTU sequences in FASTA format.

**Data S2.** Summary of pyrosequencing reads that passed quality filtering.

**Data S3.** 836 OTUs observed in the root samples.

**Data S4.** Matrix representing the presence/absence of fungal OTUs in each root sample.

**Data S5.** Matrix representing the symbiosis of plant species and fungal OTUs.

**Figure S1.** Diversity of host plants and fungi in the samples. (A) Composition of host plant species identified by chloroplast *rbcL* sequences. The number of root samples is given in parentheses. (B) Phylum-level composition of fungal OTUs observed in root samples (676 of 836 OTUs were assigned at the phylum level). (C) Order-level composition of fungal OTUs observed in root samples (431 of 836 OTUs were assigned at the order level). (D) Genus-level composition of fungal OTUs observed in root samples (221 of 836 MOTUs were assigned at the genus level).

**Figure S2.** Rarefaction curves of fungal OTUs against the numbers of sequencing reads and samples. (A) Rarefaction curve of fungal OTUs in each terminal root sample against the number of pyrosequencing reads excluding singletons. (B) Rarefaction curve of fungal OTUs against sample size. The shaded area represents the standard deviation (standard error of the estimate) obtained from 100 randomizations of sample order.

**Figure S3.** Host-specific and generalist fungi shared between pairs of dominant plant species. In each pair of the five most dominant plant species (Fig. S1A), a CLAM analysis (Chazdon et al. 2011) classified fungal OTUs into the following categories: fungi common on both plants (circle), fungi preferentially associated with either plant (square and diamond), and fungi that were too rare to be assigned association specificity (triangle). The OTU IDs of fungi with significant host preference are indicated under the symbols. For simplicity, results of the pairs of the five most common plant species in the community (Fig. S1A) are shown (see also Fig. 3). (A) *Quercus glauca* versus *Lyonia ovalifolia*. (B) *Pinus densiflora* versus *Q. glauca*. (C) *L. ovalifolia* versus *Q. serrata*. (D) *Pinus densiflora* versus *Q. serrata*. (E) *Ilex pedunculosa* versus *L. ovalifolia*. (F) *I. pedunculosa* versus *P. densiflora*. (G) *L. ovalifolia* versus *P. densiflora*.

**Table S1.** The *d*′ index for respective plant species and fungal OTUs.

**Table S2.** Statistically significant specialists and generalists revealed by CLAM test.
