# Supplementary figures and images for: How are plant and fungal communities linked to each other in belowground ecosystems? A massively parallel pyrosequencing analysis of the association specificity of root-associated fungi and their host plants
Source: Ecol Evol. 2013 Aug 2;3(9):3112–24. doi: 10.1002/ece3.706 (PMC3790555; doi:10.1002/ece3.706)

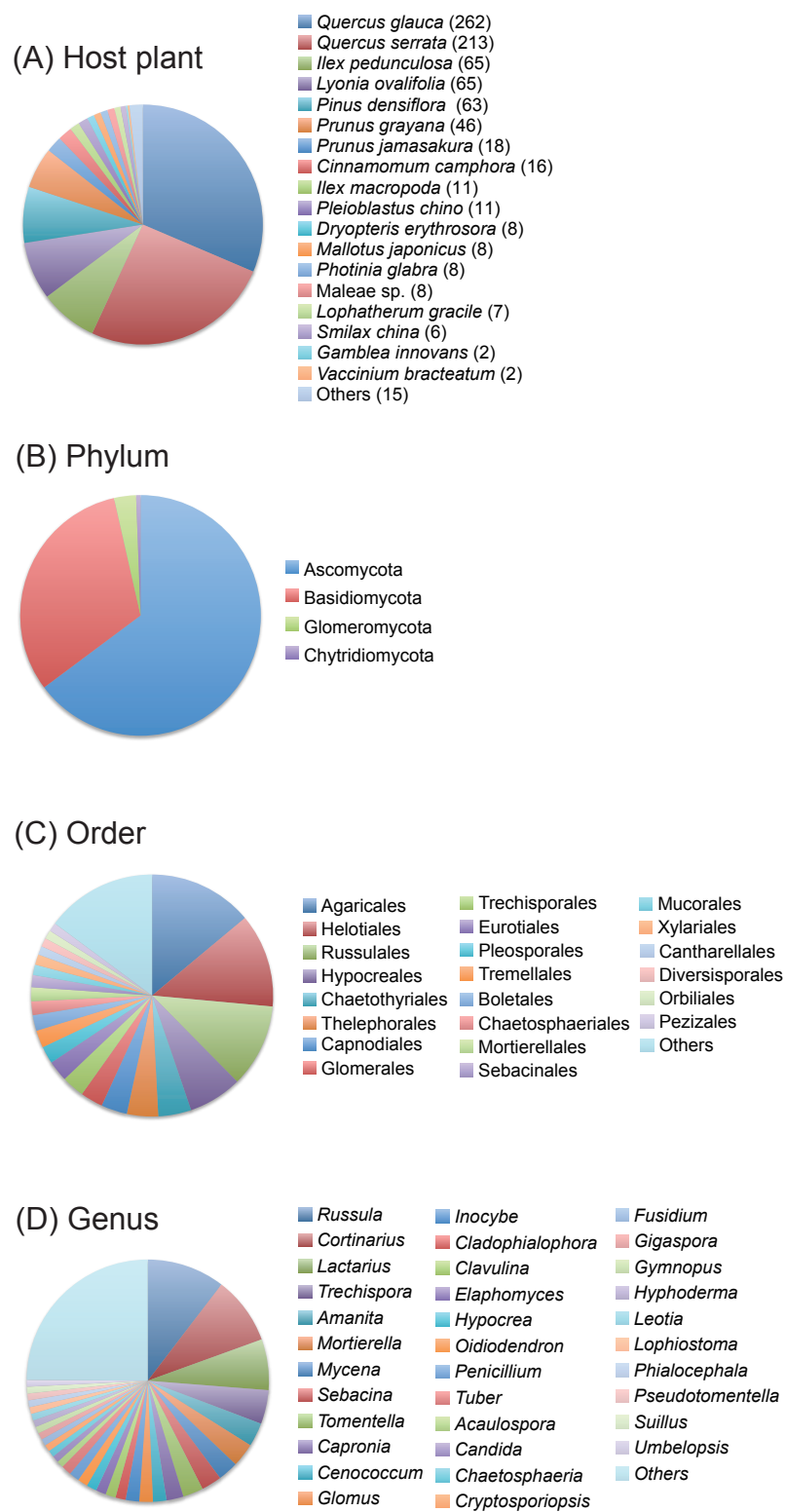

Fig. S1

Supplement: Supplementary file 6 [file ece30003-3112-SD6.pdf]

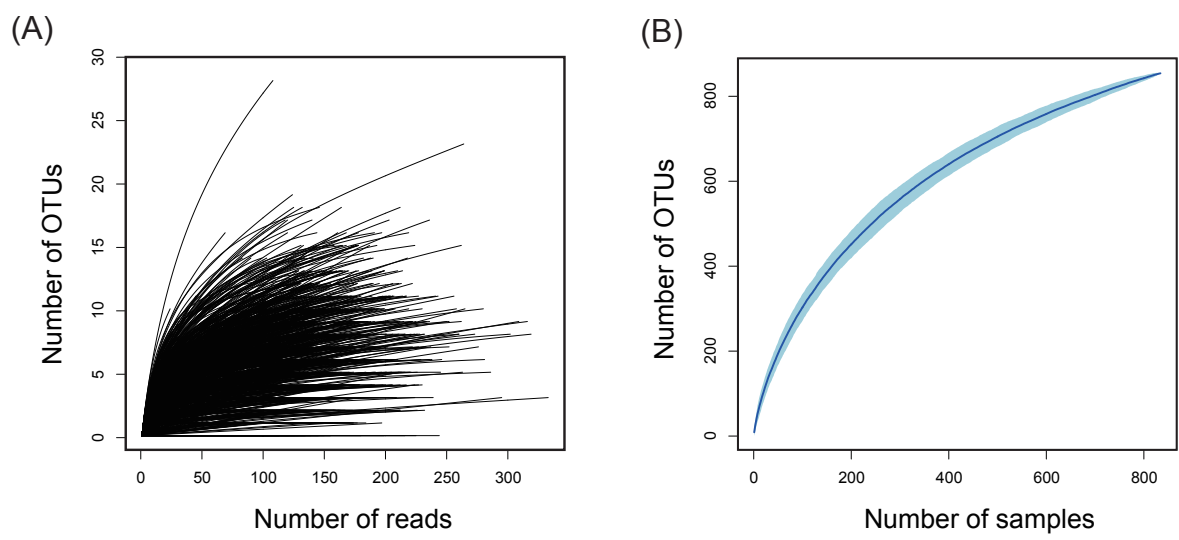

Fig. S2

Supplement: Supplementary file 7 [file ece30003-3112-SD7.pdf]

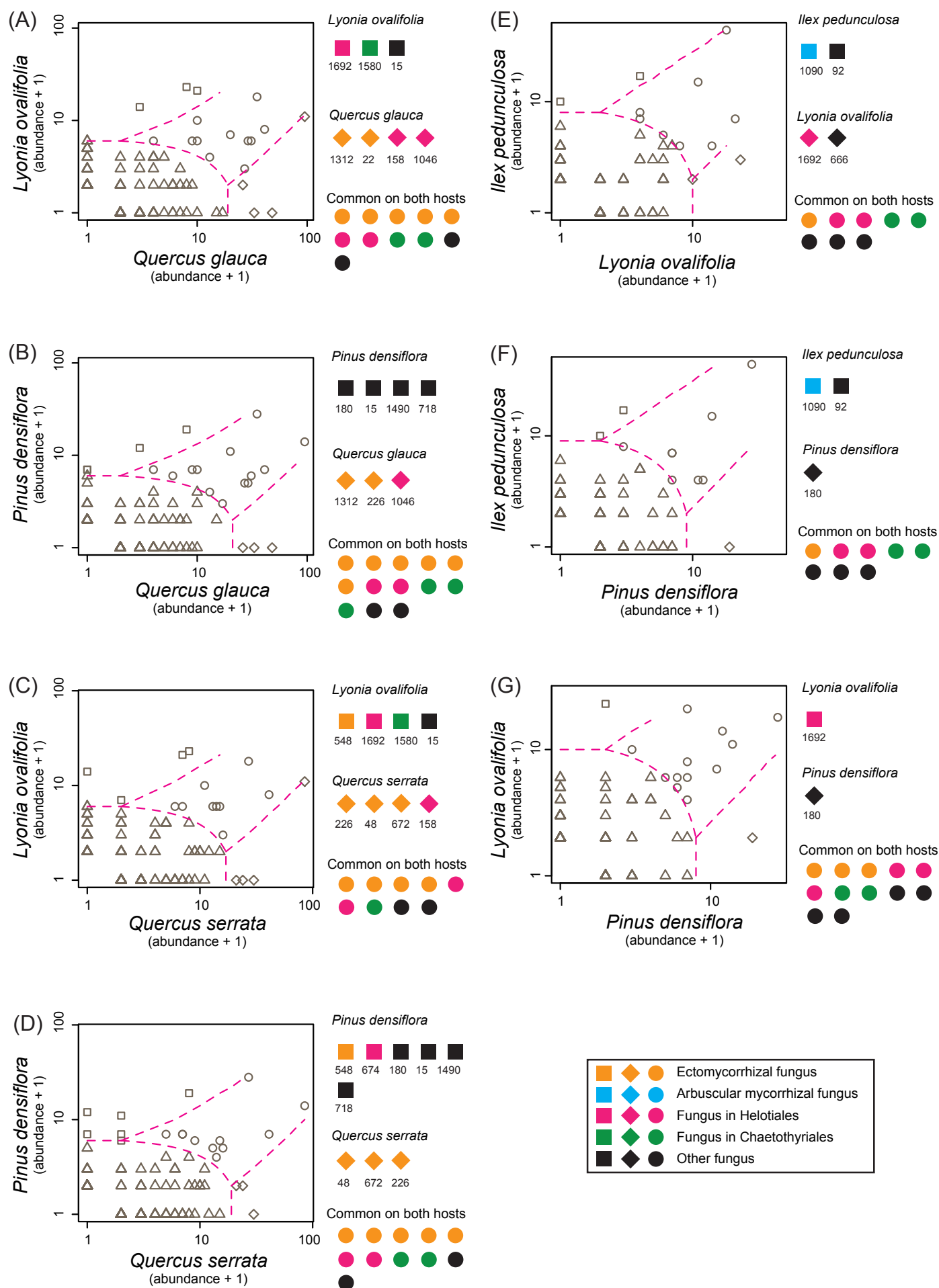

Fig. S3

Supplement: Supplementary file 8 [file ece30003-3112-SD8.pdf]
